# Smooth versus Textured Tissue Expanders: Comparison of Outcomes and Complications in 536 Implants

**DOI:** 10.1055/s-0043-1775592

**Published:** 2024-02-07

**Authors:** Omar Allam, Jacob Dinis, Mariana N. Almeida, Alexandra Junn, Mohammad Ali Mozaffari, Rema Shah, Lauren Chong, Olamide Olawoyin, Sumarth Mehta, Kitae Eric Park, Tomer Avraham, Michael Alperovich

**Affiliations:** 1 Division of Plastic Surgery, Department of Surgery, Yale School of Medicine, New Haven, Connecticut

**Keywords:** breast reconstruction, tissue expanders, complications

## Abstract

**Background** Increasing concerns regarding the safety of textured surface implants have resulted in surgeons transitioning from textured tissue expanders (TEs) to smooth TEs. Given this change has only recently occurred, this study evaluated outcomes between smooth and textured TEs.

**Methods** Women who underwent two-stage breast reconstruction using TEs from 2013 to 2022 were included. TE-specific variables, perioperative information, pain scores, and complications were collected. Chi-squared, *t* -test, and linear regression analyses were performed.

**Results** A total of 320 patients received a total of 384 textured and 152 smooth TEs. Note that 216 patients received bilateral reconstruction. TEs were removed in 9 cases. No significant differences existed between groups regarding comorbidities. Smooth TEs had a higher proportion of prepectoral placement ( *p* < 0.001). Smooth TEs had less fills (3 ± 1 vs. 4 ± 2, *p* < 0.001), shorter expansion periods (60 ± 44 vs. 90 ± 77 days, *p* < 0.001), smaller expander fill volumes (390 ± 168 vs. 478 ± 177 mL, *p* < 0.001), and shorter time to exchange (80 ± 43 vs. 104 ± 39 days, *p* < 0.001). Complication rates between textured and smooth TEs were comparable. Smooth TE had a greater proportion of TE replacements ( *p* = 0.030). On regression analysis, pain scores were more closely associated with age ( *p* = 0.018) and TE texture ( *p* = 0.046). Additional procedures at time of TE exchange ( *p* < 0.001) and textured TE ( *p* = 0.017) led to longer operative times.

**Conclusion** As many surgeons have transitioned away from textured implants, our study shows that smooth TEs have similar outcomes to the textured alternatives.

## Introduction

Postmastectomy reconstruction rates have increased to 60% among cancer patients with a growing trend toward increased implant-based reconstruction (IBR). 1 2 Patients undergoing IBR have high satisfaction as assessed with the BREAST-Q. 3 4 Protection for patients through the Women's Health and Cancer Rights Act of 1998 and recent expansion of insurance nationwide have provided access for reconstruction to more women. 5 6

Although direct-to-implant reconstruction has increased recently, most IBR is still performed with a two-stage tissue expander (TE) implant reconstruction. 7 The majority of TEs have historically had a textured surface. A textured surface is beneficial for maintaining the TE position and reducing the risk of rotation and displacement during expansion. Textured implants and TEs were historically believed to reduce capsular contracture rates as well. 8

Recent concerns regarding the safety of textured surface implants have led to a shift away from textured permanent implants and, in some cases, TEs. 9 10 Considering that smooth TEs have been in use for a limited period of time, there is a paucity of information evaluating smooth expanders' outcomes. 11

In response, this study sought to compare the reconstructive outcomes between smooth and textured TEs. Expansion time and schedule, postoperative complications, and revisionary surgeries were compared between smooth and textured TEs.

## Methods

Following Institutional Review Board approval (HIC no.: 2000221587), this study was performed in accordance with the ethical standards as laid down in the 1964 Declaration of Helsinki and its later amendments or comparable ethical standards. A retrospective chart review was conducted for women who underwent two-stage breast reconstruction using Mentor TEs from 2013 to 2022. Demographic variables collected included age, body mass index (BMI), race, the American Society of Anesthesiologists (ASA) class, comorbidities, smoking status, history of abdominal surgery, cancer type and stage, and history of chemotherapy or radiation.

TE-specific variables collected included the type of expander (Artoura, CPX4, CPX3, and CPX2), smooth versus textured surface, prepectoral versus subpectoral plane, use of acellular dermal matrices, number of total fills, final fill volume, and time until final TE fill. Additional perioperative information collected included antibiotic use, total number of days until drain removal, and pain score during the hospital stay.

Both 30-day and all complications were queried including wound infection, wound dehiscence, hematoma, seroma, return to the operating room, TE replacement, and capsular contractures. Furthermore, the need and type of any revisionary procedures as well as total operative time at TE to implant exchange procedure were recorded. A chi-square assessment, analysis of variance (ANOVA), Student's *t* -test, linear, and logistic regression analysis were conducted using SPSS Statistics (IBM, Boston, MA, 2015). Propensity score analysis was conducted to reduce the differences between TE groups by baseline characteristics. Propensity scores were based on demographic variables, such as age, BMI, and race, and comorbidities, such as diabetes. Propensity score weights were included in a weighted logistic regression analysis to evaluate the effects of TE type on complication rates. Significance was set as *p* < 0.05.

## Results

### Patient Demographics

Over 10 years, 320 patients received TEs with 384 textured and 152 smooth TEs. Mean age was 49 ± 12 years and mean BMI was 27 ± 6 kg/m ^2^ . One hundred and four patients received unilateral reconstruction, while 216 received bilateral reconstruction. There were no significant differences between groups with respect to demographics including age, BMI, or the ASA class ( Table 1 ). The two groups had comparable comorbidities with respect to rates of diabetes, hypertension, and smoking status ( Table 2 ). Among the cancer characteristics, the two groups were similar across history of radiation and chemotherapy, and cancer stage. There were more patients who had invasive ductal carcinoma who received smooth TEs compared with textured TEs (43% vs. 35%, *p* = 0.003; Table 3 ).

**Table 1:** 

	All ( *n* = 536)	Textured ( *n* = 384)	Smooth ( *n* = 152)	*p* -Value
Mean age (y)	49 ± 12	49 ± 11	48 ± 12	0.948 a
Mean BMI (kg/m ^2^ )	27 ± 6	27 ± 6	26 ± 7	0.304 a
Race				** 0.011 b**
Caucasian	391 (73%)	288 (75%)	103 (68%)	
Hispanic	55 (10%)	42 (11%)	13 (9%)	
African American	69 (13%)	40 (10%)	29 (20%)	
Asian	12 (2%)	10 (3%)	2 (1%)	
Other	8 (2%)	3 (1%)	5 (3%)	
ASA class				0.543 b
1	12 (2%)	10 (3%)	2 (1%)	
2	315 (59%)	228 (59%)	87 (57%)	
3	209 (39%)	146 (38%)	63 (41%)	

Abbreviations: ASA, American Society of Anesthesiologists; BMI, body mass index.

Note: Bold *p* -values are statistically significant.

a Independent sample *t* -test.

b Chi-square test.

**Table 2:** Patient comorbidities

Comorbidities	All ( *n* = 536)	Textured ( *n* = 384)	Smooth ( *n* = 152)	*p* -Value a
Diabetes	49 (9%)	37 (10%)	12 (8%)	0.318
Hypertension	127 (24%)	90 (23%)	37 (24%)	0.471
Smoking				0.167
Nonsmoker	292 (55%)	215 (56%)	81 (53%)	
Current smoker	50 (9%)	30 (8%)	20 (13%)	
Former smoker	190 (35%)	139 (36%)	51 (34%)	

a Chi-square test.

**Table 3:** Patient cancer characteristics

	All ( *n* = 536)	Textured ( *n* = 384)	Smooth ( *n* = 152)	*p* -Value a
History of radiation	28 (5%)	23 (6%)	5 (2%)	0.146
Adjuvant radiation	66 (12%)	47 (12%)	19 (8%)	0.519
Neoadjuvant chemo	126 (24%)	85 (22%)	41 (27%)	0.144
Adjuvant chemo	158 (30%)	109 (28%)	49 (32%)	0.219
Cancer stage				0.417
0	76 (14%)	58 (15%)	18 (12%)	
1	124 (23%)	83 (22%)	41 (26%)	
2	91 (17%)	63 (16%)	28 (18%)	
3	37 (7%)	26 (7%)	11 (7%)	
4	3 (1%)	1(1%)	2 (1%)	
Prophylactic	204 (38%)	153 (39%)	52 (34%)	
Cancer type				**0.003**
DCIS	72 (13%)	56 (14%)	16 (11%)	
Invasive ductal	201 (38%)	135 (35%)	66 (43%)	
LCIS	12 (2%)	8 (2%)	4 (3%)	
Invasive lobular	33 (6%)	27 (7%)	6 (4%)	
Mixed	5 (1%)	0 (0%)	5 (3%)	
Other	6 (1%)	5 (1%)	1 (1%)	
None	208 (39%)	153 (40%)	55 (36%)	

Abbreviations: DCIS, ductal carcinoma in situ; LCIS, lobular carcinoma in situ.

Note: Bold *p* -values are statistically significant.

a Chi-square test.

### Tissue Expander-Specific Data

The specific TE type differed between the smooth and textured cohorts ( Table 4 ). The smooth TE cohort subtype had a higher proportion of Artoura TEs (textured 4% [17/386] vs. smooth 41% [62/152], *p* < 0.001), whereas the textured TE cohort had a higher proportion of CPX4 expanders (textured 77% [298/384] vs. smooth 52% [79/152], *p* < 0.001; Table 4 ). Additionally, all CPX2 and CPX3 expanders used were exclusively in textured cases as they were not produced in smooth styles.

**Table 4:** Tissue expander-specific data

	All ( *n* = 536)	Textured ( *n* = 384)	Smooth ( *n* = 152)	*p* -Value
Number of fills	4 ± 2	4 ± 2	3 ± 1	** <0.001 a**
Tissue expansion time	81 ± 70 d	90 ± 77 d	60 ± 44 d	** <0.001 a**
Additional procedures	211 (40%)	187 (49%)	24 (16%)	** <0.001 b**
Bilateral TEs	216 (68%)	148 (64%)	68 (44%)	0.301 b
Total fill volume	463 ± 179 mL	478 ± 177 mL	390 ± 168 mL	** <0.001 a**
Drain duration	17 ± 8 d	17 ± 8 d	16 ± 8 d	** 0.019 a**
Time to exchange	98 ± 42 min	104 ± 39 min	80 ± 43 min	** <0.001 a**
Pain	6 ± 3	7 ± 2	6 ± 4	0.148 a
Postop Abx use	512 (96%)	372 (97%)	140 (98%)	0.058 **b**
Tissue expander type				** <0.001 b**
Artoura	79 (15%)	17 (4%)	62 (41%)	
CPX4	377 (70%)	298 (77%)	79 (5%)	
CPX2	48 (9%)	48 (13%)	0 (0%)	
CPX3	11 (2%)	11 (3%)	0 (0%)	
Other	19 (4%)	10 (3%)	9 (6%)	
Tissue expander plane				** <0.001 b**
Prepectoral	69 (13%)	9 (2%)	60 (60%)	
Subpectoral	467 (87%)	375 (98%)	92 (40%)	
Acellular dermal matrix use	285 (53%)	174 (42%)	112 (72%)	** <0.001 b**

Abbreviations: Abx, antibiotics; TE, tissue expander.

Note: Bold *p* -values are statistically significant.

a *t* -Test.

b Chi-square.

Smooth TEs had a higher proportion of prepectoral placement (textured 2% [9/384] vs. smooth 40% [60/152] while textured TEs were more likely to be placed subpectorally (textured 98% [375/384] vs. smooth 60% [92/152], *p* < 0.001). Drain duration was longer in patients who received textured TEs (17 ± 8 vs. 16 ± 8 days, *p* = 0.019).

There was no significant difference between pain scores for smooth and textured TEs. The maximum pain score during the in-patient stay for smooth TEs was 6 ± 4 compared with 7 ± 2 for the textured TE cohort ( *p* = 0.148). Patients with smooth TEs received a reduced number of fills (4 ± 2 for textured TEs vs. 3 ± 1 for smooth TEs, *p* < 0.001) and were more likely to have a reduced expansion period (90 ± 77 days for textured TEs vs. 60 ± 44 days for smooth TEs, *p* < 0.001), reduced final expander fill volume (478 ± 177 mL for the textured TEs vs. 390 ± 168 mL for the smooth TEs, *p* < 0.001), and reduced time to exchange (104 ± 39 minutes for textured TEs vs. 80 ± 43 minutes for smooth TEs, *p* < 0.001; Table 4 ). Additionally, there were more symmetrizing mastopexy/reduction procedures completed in patients who received smooth TEs compared with patients who received textured TEs (38% vs. 26%, *p* = 0.004).

### Complication Rates between Textured and Smooth TEs

No significant differences were found in complication rates between textured and smooth TEs ( Table 5 ). Rates of infection (5 vs. 9%, *p* = 0.114), hematoma (5 vs. 2%, *p* = 0.153), seroma (5 vs. 6%, *p* = 0.832), and wound breakdown (4 vs. 6%, *p* = 1.00) were not significantly different between groups. The two cohorts had comparable rates of return to the operating room in the first 30 days (8 vs. 9%, *p* = 0.597) and similar rates of capsular contractions (21 vs. 15%, *p* = 0.145). However, there were more smooth TE replacements (4 vs. 10%, *p* = 0.030; Table 5 ). Of all 30 TE replacements, 8 (26.7%) were due to the patient's desire to remove the TE (4 textured vs. 4 smooth TE), 19 (63.3%) were due to either infections, chronic seroma, or mastectomy flap necrosis (8 textured vs. 11 smooth TEs), and 3 (10.0%) were due to TE malfunction (all textured TEs). There was no difference between smooth and textured TE regarding the reason for TE replacements ( *p* = 0.26). Furthermore, among those who received radiation ( *n* = 90), there were no differences between smooth and textured TEs for rates of infection, hematoma, seroma, wound dehiscence, return to the operating room, TE explantation, and capsular contracture.

**Table 5:** Comparison of postoperative complications between smooth and textured tissue expanders

Complications	All ( *n* = 536)	Textured ( *n* = 384)	Smooth ( *n* = 152)	*p* -Value a
Infection requiring IV Abx	34 (6%)	20 (5%)	14 (9%)	0.114
Hematoma	23 (4%)	20 (5%)	3 (2%)	0.153
Seroma	29 (6%)	20 (5%)	9 (6%)	0.832
Wound breakdown/necrosis	23 (4%)	17 (4%)	6 (4%)	1.00
Return to OR within 30 d	43 (8%)	29 (8%)	14 (9%)	0.597
TE replacement	30 (5%)	15 (4%)	15 (10%)	**0.030**
Capsular contracture	103 (19%)	80 (21%)	23 (15%)	0.145

Abbreviations: Abx, antibiotics; IV, intravenous; OR, operating room; TE, tissue expander.

Note: Bold *p* -values are statistically significant.

a Chi-square.

On further regression analysis evaluating the association between reported complications with TE type, while controlling for BMI, diabetes, TE plane placement, acellular dermal matrix, adjuvant radiation, and adjuvant chemotherapy, smooth TEs (odds ratio [OR] = 3.548, 95% confidence interval [CI]: 1.595–7.891, *p* = 0.002) were only associated with having TE replacement ( Table 6 ). The association between smooth TEs and TE replacement persisted on weighted multivariate regression utilizing propensity scores. Additionally, smooth TEs were less likely to have capsular contractures (OR = 0.657, 95% CI: 0.449–0.963, *p* = 0.031). Furthermore, after stratifying by TE model type, no significant differences were found between smooth and textured TEs regardless of whether patients received the CPX4 or Artoura subtype. These subgroups also had no effect on maximum pain score, expansion period, final expander fill volumes, and time to exchange.

**Table 6:** Multivariate regression model for complications after tissue expander placement

	Infection		Hematomas		Seromas		Wound breakdown		Return to operation room in 30 days		TE replacement		Capsular contracture	
	OR (95% CI)	*p* -Value a	OR (95% CI)	*p* -Value a	OR (95% CI)	*p* -Value a	OR (95% CI)	*p* -Value a	OR (95% CI)	*p* -Value a	OR (95% CI)	*p* -Value a	OR (95% CI)	*p* -Value a
Smooth TE vs. textured TE	1.671 (0.668–4.182)	0.273	0.566 (0.134–2.392)	0.439	1.307 (0.491–3.482)	0.592	1.369 (0.486–3.856)	0.552	1.511 (0.667–3.422)	0.323	3.548 (1.595–7.891)	**0.002**	0.739 (0.396–1.381)	0.343
BMI	0.990 (0.933–1.050)	0.735	0.917 (0.837–1.004)	0.061	0.953 (0.886–1.025	0.199	1.061 (1.000–1.127)	0.051	0.994 (0.943–1.047)	0.808	1.033 (0.981–1.087)	0.222	1.013 (0.978–1.050)	0.462
Diabetes	1.083 (0.298–3.934)	0.904	2.271 (0.590–8.748)	0.233	0.779 (0.163–3.710)	0.753	0.277 (0.34–2.252)	0.230	2.274 (0.841–6.145)	0.105	3.446 (1.354–8.767)	**0.009**	1.698 (0.828–3.479)	0.148
Subpectoral placement vs. prepectoral	0.553 (0.169–1.811)	0.328	1.009 (0.090–11.325)	0.994	1.651 (0.383–7.109)	0.501	3.923 (0.437–35.203)	0.222	2.324 (0.653–8.268)	0.193	1.713 (0.568–5.165)	0.339	0.881 (0.360–2.158)	0.781
Acellular dermal matrix	0.735 (0.314–1.716)	0.476	0.357 (0.125–1.020)	0.054	1.479 9(0.628–3.487)	0.371	1.219 (0.499–2.978)	0.663	1.353 (0.667–2.745)	0.402	1.549 (0.695–3.452)	0.284	0.684 (0.420–1.115)	0.128
Adjuvant radiation	1.996 (0.774–5.1351)	0.153	2.504 (0.814–7.701)	0.109	3.111 (1.246–7.768)	**0.015**	2.853 (1.017–8.008)	**0.046**	0.614 (0.216–1.748)	0.361	1.252 (0.453–3.463)	0.665	3.390 (1.887–6.089)	**<0.001**
Adjuvant chemotherapy	1.813 (0.844–3.895)	0.127	0.90 (0.32–2.56)	0.790	2.165 (0.951–4.928)	0.066	1.152 (0.456–2.910)	0.765	2.976 (1.502–5.895)	**0.002**	1.063 (0.481–2.352)	0.880	1.898 (1.183–3.043)	**0.008**

Abbreviations: BMI, body mass index; CI, confidence interval; OR, odds ratio; TE, tissue expander.

Note: Bold *p* -values are statistically significant.

a Multivariate logistic regression.

In a separate regression analysis, maximum pain score was closely associated with age ( *p* < 0.018; Table 7 ). Older patients had higher pain scores. Textured TEs were associated with higher pain scores than smooth TEs ( *p* = 0.046). No association was found between TE subtype and pain scores ( *p* = 0.472). Surprisingly, TE pocket (subpectoral vs. prepectoral) did not have a significant impact on pain scores ( *p* = 0.885). Having more associated procedures at the time of TE exchange ( *p* < 0.001), greater fill volume ( *p* = 0.011), and textured TE ( *p* = 0.017) led to longer operative times for TE exchange ( Table 7 ).

**Table 7:** Multivariate regression models for maximum pain scores after tissue expander placement and length of time in the operating room for exchange to implant

	Pain		Exchange OR time	
Covariates	Standardized *B*	*p* -Value a	Standardized *B*	*p* -Value a
ASA	0.252	**0.009**	–0.001	0.983
Insurance	–0.074	0.406	–0.060	0.205
Age	–0.280	**0.018**	0.103	0.059
Hypertension	0.143	0.144	0.010	0.844
Adjuvant radiation	0.006	0.950	–0.015	0.752
Adjuvant chemo	0.080	0.443	–0.011	0.818
TE type	–0.264	**0.046**	–0.162	**0.017**
Subpectoral vs. prepectoral plane	0.045	0.726	–1.000	0.159
Surgeon	0.071	0.483	0.138	**0.010**
Subtype	0.070	0.472	0.013	0.802
Additional procedures	0.014	0.892	0.265	**<0.001**
Unilateral vs. bilateral	0.179	.082	0.060	0.240
Acellular dermal matrix	–0.115	0.266	–0.078	0.135
Fill volume	–0.019	0.845	0.138	**0.011**

Abbreviations: ASA, American Society of Anesthesiologists; OR, operating room; TE, tissue expander.

Note: Bold *p* -values are statistically significant.

a Multivariate linear regression.

### Complication Rates by TE Plane Placement

We further stratified the groups based on the plane placement of the TE, prepectoral and subpectoral. There were 467 TEs that were placed in the subpectoral plane (375 textured and 92 smooth). Between the textured and smooth TEs, there were more TE replacements for smooth TEs (6% vs. 13%, *p* = 0.026). However, there were no differences in rates of infection, hematomas, seromas, wound breakdown/necrosis, return to the operating room within 30 days, and capsular contracture. Furthermore, there was no difference in pain scores (7 ± 2 vs. 6 ± 2, *p* = 0.896). In the prepectoral plane, there were 69 TEs (9 textured TEs and 60 smooth TEs). There was a greater proportion of breast hematomas in those who received textured TEs in the prepectoral planes (22% vs. 1.7%, *p* = 0.043). Compared with smooth TEs, there was no difference in rates of infection, seroma, wound dehiscence, return to the operating room, need for TE replacement, and capsular contractures ( Table 8 ). Additionally, there were no differences in reported maximum pain levels (8 ± 2 vs. 5 ± 4, *p* = 0.108).

**Table 8:** Subgroup analysis of complications between smooth and textured tissue expanders placed in the subpectoral plane and prepectoral plane

Complications	All	Textured	Smooth	*p* -Value a
Subpectoral plane, *n*	467	375	92	
Infection requiring IV Abx	24 (5%)	18 (5%)	6 (7%)	0.597
Hematoma	20 (4%)	18 (5%)	2 (2%)	0.391
Seroma	24 (5%)	18 (5%)	6 (7%)	0.597
Wound breakdown/necrosis	22 (5%)	17 (5%)	5 (5%)	0.783
Return to OR within 30 days	37 (8%)	27 (7%)	10 (11%)	0.281
TE replacement	24 (6%)	15 (6%)	9 (13%)	**0.024**
Capsular contracture	93 (%)	79 (21%)	14 (15%)	0.245
Prepectoral plane, *n*	69	9	60	
Infection requiring IV Abx	11 (16%)	2 (22%)	9 (15%)	0.333
Hematoma	3 (4%)	2 (22%)	1 (2%)	**0.043**
Seroma	5 (7%)	2 (22%)	3 (5%)	0.124
Wound breakdown/necrosis	1 (1%)	0 (0%)	1 (2%)	0.087
Return to OR within 30 days	6 (9%)	2 (22%)	4 (7%)	0.172
TE replacement	6 (9%)	0 (0%)	6 (10%)	0.306
Capsular contracture	10 (14%)	1 (11%)	9 (15%)	1.000

Abbreviations: Abx, antibiotics; IV, intravenous; OR, operating room; TE, tissue expander.

Note: Bold *p* -values are statistically significant.

a Chi-square.

We further stratified patients who did and did not have any exposure to radiation. Of the patients who were exposed to any radiation, there were 83 TEs placed in the subpectoral plane (67 textured and 21 smooth) and 7 TEs placed in the prepectoral plane (2 textured and 5 smooth). Among the subpectoral plane, there were no differences in complication rates between textured and smooth TE. Among the prepectoral plane, there were hematomas and seromas found among the two textured TEs, while no hematomas and seromas were found in the 5 smooth TEs ( *p* = 0.048). There were no significant differences in infection rates, wound dehiscence, return to the operating room, TE replacement, and capsular contractures. Of the patients who were not exposed to radiation, there were 384 TEs placed in the subpectoral plane (308 textured and 76 smooth). There were more returns to the operating room within 30 days (13 vs. 5%, *p* = 0.041) and TE replacements (10 vs. 2%, *p* < 0.001) for smooth TEs compared with textured TEs. There was no difference in rates for infection, hematomas, seromas, wound breakdown/necrosis, or capsular contracture. There were 62 TEs that were placed in the prepectoral plane (7 textured and 55 smooth). However, there were no incidences of infections, hematomas, seromas, and wound breakdown/necrosis for patients who received textured TEs. Additionally, there was no difference in rates of capsular contractions ( Table 9 ).

**Table 9:** Subgroup analysis of complications between smooth and textured tissue expanders place in the subpectoral plane and prepectoral plane in patients based on radiation exposure

Complications	All	Textured	Smooth	*p* -Value a
Radiation exposure, *n*	90	69	21	–
Subpectoral plane, *n*	83	67	16	–
Infection requiring IV Abx	7 (8%)	6 (9%)	1 (6%)	1.00
Hematoma	6	6 (9%)	0 (0%)	0.591
Seroma	12	9 (13%)	3 (19%)	0.693
Wound breakdown/necrosis	8	7 (10%)	1 (6%)	1.00
Return to OR within 30 days	10	10 (15%)	0 (0%)	0.197
TE replacement	9	8 (12%)	1 (6%)	1.00
Capsular contracture	32	29 (43%)	3 (19%)	0.090
Prepectoral plane, *n*	7	2	5	–
Infection requiring IV Abx	3	2 (100%)	1 (20%)	0.053
Hematoma	2	2 (100%)	0 (0%)	**0.048**
Seroma	2	2 (100%)	0 (0%)	**0.048**
Wound breakdown/necrosis	1	0 (0%)	1 (20%)	1.00
Return to OR within 30 days	3	2 (100%)	1 (20%)	0.143
TE replacement	0	0 (0%)	0 (0%)	–
Capsular contracture	2	0 (0%)	2 (40%)	1.00
No radiation exposure, *n*	446	315	131	
Subpectoral plane, *n*	384	308	76	
Infection requiring IV Abx	18 (5%)	12(4%)	6 (7%)	0.348
Hematoma	14 (4%)	12 (4%)	2 (3%)	1.00
Seroma	15 (5%)	9 (3%)	6 (8%)	0.890
Wound breakdown/necrosis	15 4(%)	10 (3%)	5 (6%)	0.101
Return to OR within 30 days	27 (7%)	17 (5%)	10 (13%)	**0.041**
TE replacement	21 (5%)	7 (2%)	8 (10%)	**<0.001**
Capsular contracture	61 (16%)	51 (17%)	10 (13%)	0.599
Prepectoral plane, *n*	62	7	55	–
Infection requiring IV Abx	6 (9%)	0 (0%)	6 (10%)	1.00
Hematoma	1 (2%)	0 (0%)	1 (2%)	1.00
Seroma	3 (5%)	0 (0%)	3 (5%)	1.00
Wound breakdown/necrosis	0 (0%)	0 (0%)	0 (0%)	–
Return to OR within 30 days	3 (5%)	0 (0%)	3 (5%)	1.00
TE replacement	6 (9%)	0 (0%)	6 (10%)	0.580
Capsular contracture	8 (13%)	1 (14%)	7 (12%)	1.00

Abbreviations: Abx, antibiotics; IV, intravenous; OR, operating room; TE, tissue expander.

Note: Bold *p* -values are statistically significant.

a Chi-square.

## Discussion

Immediate breast reconstruction has continued to grow in popularity with a well-described improvement in quality of life compared with postmastectomy alternatives. 12 13 Following recent concerns regarding the long-term safety of textured implants, many plastic surgeons have transitioned to predominantly using smooth implants and TEs. While smooth implants have been used with high frequency, limited data exist regarding the efficacy of smooth TEs. 7 This study provides an objective comparison between smooth and textured TEs by expander fill characteristics, complications, and pain.

In the two well-matched cohorts, there were no significant differences in complication rates between smooth and textured TEs. A previous study identified that the Mentor textured TE tends to adhere less to soft tissue compared with other brands of textured TEs. Given this, the Mentor textured TE may function like the smooth TEs. 14 The similarities between the two TEs may contribute to the comparable characteristics found in this present study between the smooth and textured TEs.

Although radiation exposure may influence the rates of infection and dehiscence/necrosis, there were no differences between smooth and textured TEs when evaluating patients with and without radiation exposure in our cohort. The higher rates of hematoma and seromas among the TEs placed in the prepectoral plane in patients that received radiation exposure should be evaluated cautiously given the small amount of TEs placed in the prepectoral plane. The rates of infection in our study contrast with previous studies, which have identified higher rates of infection and explantation in textured TEs and higher seroma formation in smooth TEs. 15 16 In these studies, textured TEs were accompanied by higher rates of acellular dermal matrices usage, which have been shown to increase biofilm formation and subsequently infection and explantation. 17 Interestingly, smooth TE were associated with more replacements. The majority of the smooth TEs that were replaced were due to either an infection, seroma, or mastectomy necrosis. However, there were no differences observed between smooth and textured TEs regarding the reason behind TE replacements. Furthermore, although there were more replacements for smooth TEs, the rates of complications were similar to textured TEs.

While plane of placement may serve as a potential confounding factor, prior literature comparing prepectoral and subpectoral planes in larger cohorts of textured implants found that complication rates are comparable between the two planes. 18 19 In our study, there was no difference in complication rates by TE plane placement with the exception of more hematomas among textured TEs in the prepectoral plane. This finding should be cautiously considered given the limited number of textured TEs in the prepectoral plane ( *n* = 9) in comparison to smooth TEs ( *n* = 60). However, these differences should warrant further study when weighing the risks of textured TEs, such as breast implant-associated anaplastic large cell lymphoma. 9 10

While smooth TEs showed a reduced expansion time, these findings correspond with smaller final fill volume likely as a result of surgeon preference and preferred aesthetic technique. This is further supported by a higher proportion of patients with smooth TEs also receiving symmetrizing mastopexy/reductions compared with patients with textured TEs, indicating different outcome preferences. Interestingly, smooth versus textured TE type did influence total operating room time with smooth TEs having shorter documented operative time for implant exchange. This finding persisted even after controlling for subpectoral versus prepectoral placement, fill volume, adjunct procedures such as fat grafting, and TE subtype. Furthermore, smooth TEs had a shorter drain duration compared with textured TEs. Typically, timing of drain removal is based on output; however, it may also depend on provider practices.

There was no difference in the maximum pain levels between smooth and textured TEs. However, smooth TEs were associated with lower pain scores when controlling for other factors such as adjuvant radiation, adjuvant chemo, plane placement, among other. However, given the extended time frame of this study, there were 11 different breast surgeons that completed the mastectomy prior to TE placement. Between both smooth and textured groups, there were differences in the surgeons that performed the surgery. Given these differences, different techniques may have been completed which may have contributed to the pain differences seen between smooth TEs and textured TEs. Additionally, older patients were associated with higher pain scores, which is consistent with prior literature. 20 21 22 Although adjuvant treatments, perioperative variables, and comorbidities were controlled, the different postoperative course trajectory between patients may have played a role in the association between older patients reporting higher pain scores. Despite recent literature supporting that prepectoral TE placement results in lower pain scores than subpectoral, this did not hold true in our analysis. 23 24 Even when stratifying for plane placement, there remained no differences in the maximum pain levels experienced between the smooth and textured TEs. Given the length of time of this study, these findings are likely influenced by the lack of a standardized pain regimen and a more regulated pathway for pain management may have since been adopted.

Limitations of this study include the retrospective nature of this study. Some of the differences between cohorts reflect an evolution in technique at our institution as well as nationwide with a greater preference for prepectoral placement and Artoura over the CPX4 TE device. Furthermore, our findings represent the outcomes of our academy, a relatively higher volume institution, and may not be representative of other surgeons' experiences. Finally, due to the recent transition to smooth TEs, the total number of smooth TE was more limited compared with textured TEs. Additionally, the total number of prepectoral texture TEs was limited and given the recent transition to smooth TEs, we are unable to have even numbers of TE type between these planes. However, to evaluate the potential effect of implant plane placement we included a separate subanalysis evaluating the complication rates between textured and smooth TEs stratified by plane placement. We further controlled for plane placement in the evaluation of TE type with each complication rate to reduce any possible effects of plane placement. Future study is needed to reexamine findings in a larger cohort with longer follow-up of complications, including displacement rates. Additionally, future study is needed to evaluate the long-term aesthetic outcomes and patient-reported satisfaction.

Smooth TEs have comparable complication rates as textured TEs. Although differences in fill characteristics were seen, this may reflect different outcome and provider preference rather than distinct expander differences. Adoption of smooth TEs has mirrored the acceptance and greater use of a prepectoral implant plane. As many surgeons have transitioned away from textured implants, our study is the first to show that smooth TEs have similar outcomes to the textured alternatives.
